# Mitophagy: insights into its signaling molecules, biological functions, and therapeutic potential in breast cancer

**DOI:** 10.1038/s41420-024-02226-6

**Published:** 2024-10-29

**Authors:** Cong Chen, Aizhai Xiang, Xia Lin, Jufeng Guo, Jian Liu, Shufang Hu, Tao Rui, Qianwei Ye

**Affiliations:** Department of Breast Surgery, Affiliated Hangzhou First People’s Hospital, School of Medicine, Westlake University, Hangzhou, China

**Keywords:** Breast cancer, Mitophagy

## Abstract

Mitophagy, a form of selective autophagy that removes damaged or dysfunctional mitochondria, plays a crucial role in maintaining mitochondrial and cellular homeostasis. Recent findings suggest that defective mitophagy is closely associated with various diseases, including breast cancer. Moreover, a better understanding of the multifaceted roles of mitophagy in breast cancer progression is crucial for the treatment of this disease. Here, we will summarize the molecular mechanisms of mitophagy process. In addition, we highlight the expression patterns and roles of mitophagy-related signaling molecules in breast cancer progression and the potential implications of mitophagy for the development of breast cancer, aiming to provide better therapeutic strategies for breast cancer treatment.

## Facts

- Mitophagy removes damaged or dysfunctional mitochondria and plays a crucial role in maintaining mitochondrial homeostasis.
- Mitophagy-related signaling molecules are abnormally expressed in breast cancer and are involved in cancer biology.
- Dysregulation of mitophagy has been implicated in various diseases, including breast cancer progression.
- The modulation of mitophagy has novel therapeutic potential for breast cancer treatment.

## Open questions

- Does mitophagy increase or decrease the risk of developing breast cancer?
- In the context of different stages, different molecular types, and different tumor microenvironments of breast cancer, how does mitophagy govern the fate of breast cancer cells (promote or inhibit tumors)?
- How can adverse effects be weighed and avoided when mitophagy is modulated as a therapeutic approach in breast cancer treatment?

## Introduction

Breast cancer is the leading cause of cancer morbidity and mortality in women around the world [1]. Although modern treatments are multimodal and there have been recent advancements in therapeutics, drug resistance is common and remains a major clinical challenge in breast cancer treatment [2, 3]. Therefore, identifying effective therapeutic targets is still an important issue that needs to be solved in clinical practice. Recent studies have indicated that dysfunction of regulated cell death (RCD) is a hallmark feature of cancer and contributes to the mechanisms of tumor progression and drug resistance [4, 5]. Moreover, several new forms of RCD, such as autophagy, ferroptosis, pyroptosis, and cuproptosis, have been extensively studied and may represent promising avenues for enhancing treatment efficacy and overcoming drug resistance in breast cancer [6–9].

Mitochondrial autophagy, also known as mitophagy, is a form of selective autophagy that serves to maintain mitochondrial and cellular homeostasis. The term “mitophagy” was first proposed in 2005 [10], and subsequently, the classic process of mitophagy was characterized by significant discoveries, particularly in terms of understanding its molecular mechanisms and implications for health and disease [11]. Mitophagy is responsible for the selective degradation and removal of damaged or dysfunctional mitochondria through lysosomes [12]. This process is tightly regulated by a complex network of signaling pathways and molecules. However, uncontrolled mitophagy disrupts the basal mitochondrial turnover and leads to excessive degradation of mitochondria [13].

Recently, accumulating evidence has shown that mitophagy dysregulation has been implicated in various diseases [14, 15], including breast cancer. However, the process and roles of mitophagy in breast cancer development and progression appear to be complex, as mitophagy acts as both a tumor suppressor and a tumor promoter depending on the context, microenvironment, or stage of the cancer. Moreover, the potential of modulating mitophagy as a therapeutic strategy in breast cancer treatment remains to be further addressed. This review aims to present a current overview of the expression patterns and roles of mitophagy-related signaling molecules in breast cancer progression and the implications of mitophagy for the development and treatment of breast cancer.

## Mechanisms of mitophagy in brief

### Ubiquitin-dependent pathways

Currently, PINK1/Parkin-driven mitophagy is the most characterized pathway. In normal mitochondria, PINK1 is continuously imported into the inner membrane of the mitochondria and then cleaved by the mitochondrial proteases MPP and PARL [16, 17]. However, in the context of mitochondrial damage or depolarization, the import of PINK1 into the inner membrane is blocked, resulting in PINK1 accumulation on the outer mitochondrial membrane (OMM) [18]. This accumulation of PINK1 on the OMM leads to its stabilization and activation and subsequent phosphorylation of several substrates, including ubiquitin (Ub) and E3 ubiquitin ligases [19, 20]. Parkin, a cytosolic E3 ubiquitin ligase, can be phosphorylated at Ser65 by PINK1, which recruits more Parkin to mitochondria and activates its E3 ligase activity to generate more ubiquitin chains [21]. Subsequently, autophagy receptor proteins, including p62, TAX1BP1, NDP52, NBR1, and OPTN, contain both ubiquitin-binding domains (UBDs) that recognize phosphorylated ubiquitin chains, and LC3-interacting regions (LIRs) that facilitate interaction with LC3 family proteins, thereby initiating the autophagosome biogenesis machinery [13]. In addition to Parkin, several other ubiquitin E3 ligases, such as SMURF1, Gp78, MUL1, SIAH1, and ARIH1, also participate in the ubiquitination of mitochondrial proteins and induce mitophagy [14, 21].

### Ubiquitin-independent pathways

In addition to ubiquitin-dependent mitophagy, mitochondrial surface proteins can also serve as receptors for mitophagy and facilitate ubiquitin-independent mitophagy. These proteins contain LIRs within their structure, enabling direct binding to LC3 and/or GABARAP (GABA-receptor-associated protein) without ubiquitination, thus initiating mitophagy. BNIP3 (BCL2 interacting protein 3) is an OMM protein, and phosphorylation of Ser17 and Ser24 within its LIR motif is essential for binding to LC3 [22, 23]. BNIP3L (NIP3-like protein X, or NIX) is highly homologous to BNIP3, and phosphorylation at Ser34 and Ser35 within its LIR motif significantly increases its binding affinity to LC3 [11]. Similar to BNIP3 and BNIP3L, the phosphorylation and dephosphorylation of FUNDC1 (FUN14 domain-containing protein 1) are critical for regulating its interaction with LC3 [11]. It was reported that phosphorylation of FUNDC1 at Ser17 by ULK1 enhances its interaction with LC3, whereas dephosphorylation of FUNDC1 at Ser13 by PGAM5 facilitates its binding to LC3, thereby promoting mitophagy [24]. Attractively, an increasing number of autophagy receptor proteins, including BCL2L13, FKBP8, AMBRA1, MCL-1, SAMM50, and others, have been identified and documented in the previous literature [11, 25].

**Fig. 1:**
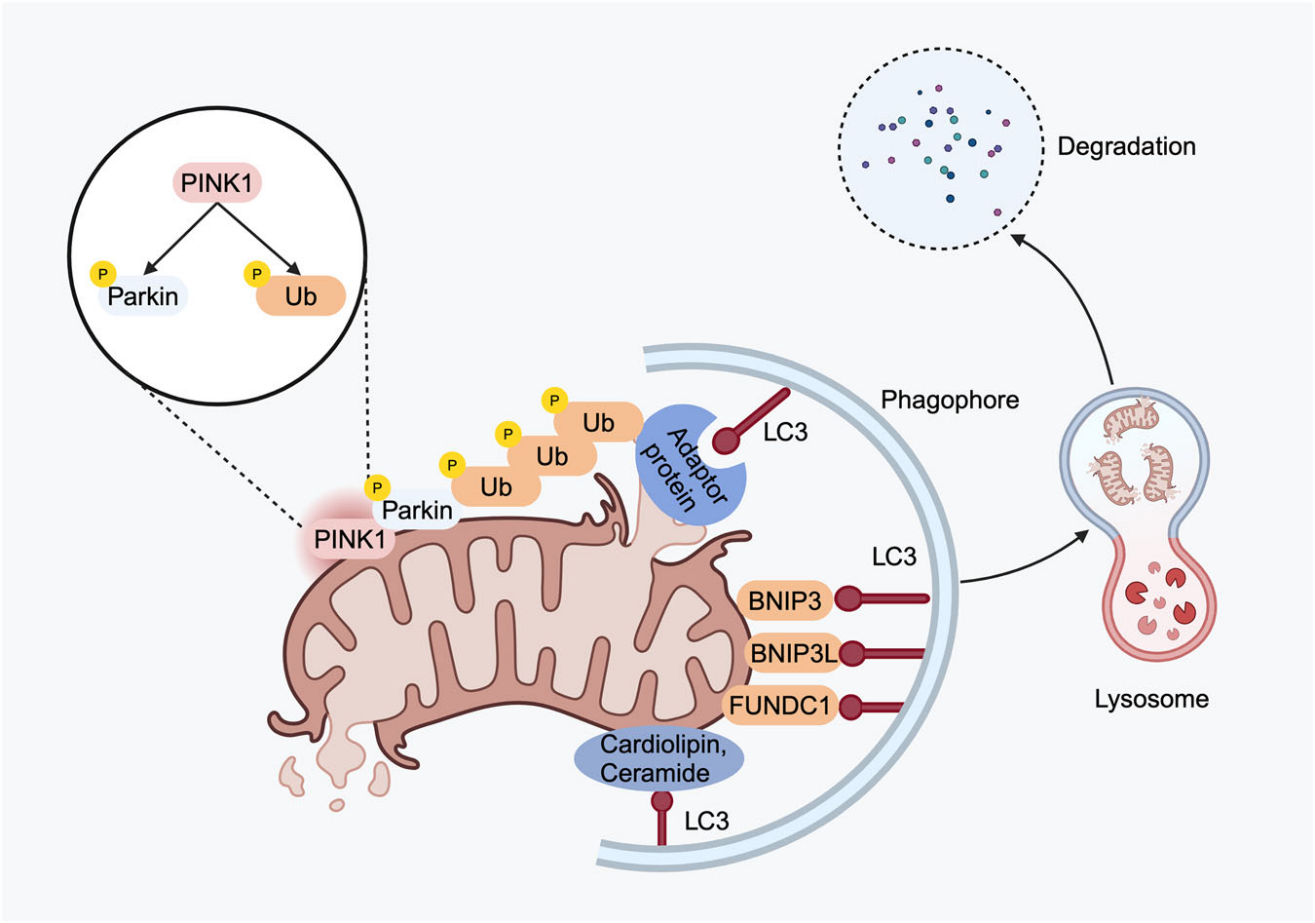
The main molecular mechanism of mitophagy. The PINK1/Parkin-driven mitophagy is the most extensively analyzed pathway. When PINK1 is stabilized and activated on the OMM, it phosphorylates ubiquitin at serine 65 and Parkin within its ubiquitin-like domain. Subsequently, autophagy receptor proteins recognize phosphorylated Ub chains and initiate autophagosome biogenesis machinery through binding with LC3. Moreover, mitochondrial surface proteins, such as BNIP3, BNIP3L, FUNDC1, and other autophagy receptor proteins, can interact directly with LC3, thus facilitating non-ubiquitin-dependent mitophagy. Additionally, certain lipids, including cardiolipin and ceramide, can also interact with LC3 and mediate lipid-dependent mitophagy. Created with BioRender.com.

In addition to protein receptors, emerging evidence indicates that certain lipids can also interact with LC3 and mediate lipid-dependent mitophagy. For example, cardiolipin (CL), primarily localized to the inner mitochondrial membrane (IMM) in healthy mitochondria, can translocate to the OMM in response to mitochondrial stress or damage. This translocation of CL facilitates direct binding to LC3 and promotes CL-mediated mitophagy [26, 27]. Additionally, ceramide, a bioactive sphingolipid, has been shown to directly bind LC3 at the OMM. This ceramide-LC3 interaction at the OMM facilitates the recruitment of autophagosomes to mitochondria and subsequently initiates mitophagy [28]. The main pathways involved in mitophagy are summarized and presented in Fig. 1.

## Major mitophagy proteins in breast cancer

### PINK1 in breast cancer

PTEN-induced kinase-1 (PINK1), a serine/threonine kinase, has been extensively studied in autosomal recessive familial Parkinson’s disease (PD) [29]. However, emerging evidence indicates that PINK1 has roles in other human diseases, such as cancers [30]. In breast cancer, Yaghoobi et al. compared the transcript levels of PINK1 in 54 breast cancer samples as well as their paired nearby tissues and reported that PINK1 mRNA expression was significantly downregulated in tumors compared with neighboring normal tissues [31]. Inconsistently, Li et al. conducted immunohistochemical (IHC) analysis on 150 cases of breast cancer tissues and 18 cases of benign specimens [32] and found a higher level of PINK1 protein expression in breast cancer tissues than in benign specimens [32]. This difference is likely because of the different nature of the tissues or the posttranscriptional regulation of PINK1. More specifically, Berthier et al. first described the subcellular distribution of the PINK1 protein in normal and tumor tissues [33]. Although PINK1 is positively expressed in both normal breast tissue and breast cancer tissue, PINK1 exhibits diffuse cytoplasmic staining along with strong membrane staining in breast cancer, whereas, in normal breast tissue, it shows a granular cytoplasmic pattern and minimal membrane staining [33]. This distinctive staining pattern indicated that the localization of PINK1 might be a new biomarker in breast cancer.

Even induced by the multifunctional tumor suppressor PTEN [34], the biological function of PINK1 in tumors remains controversial. In breast cancer, both the mitochondrial and nonmitochondrial localization of PINK1 have been shown to suppress MCF-7 cell (a luminal A cell line) growth in vitro [33]. In addition, PINK1 also confers resistance to apoptosis triggered by mitochondria-dependent apoptotic death stimuli (H_2_O_2_ treatment) [33]. This suggests that PINK1 may play multifaceted roles in tumor biology beyond its role in mitochondrial functions. Consistently, Miyahara et al. reported that PINK1 inhibition induced the expression of BRCA1, thus promoting MCF-7 cell growth [35]. Conversely, another study revealed that the absence of PINK1 inhibits MDA-MB-231 cell growth [32]. This observation supports the tumor-promoting properties of PINK1 in triple-negative breast cell (TNBC) lines. Likewise, in the HER-2 positive breast cancer subtype, inhibition of PINK1 expression has been found to enhance paclitaxel-induced apoptosis in BT-474 breast cancer cells [36]. These findings indicate that PINK1 may exert both tumor-promoting and tumor-restrictive effects on different subtypes of breast cancer cells, but the underlying mechanisms remain unclear. Moreover, cytosolic PINK1 is involved in the PI3K/Akt pathway via the activation of Akt phosphorylation [37, 38]. Since PI3K/Akt signaling is abnormally activated in breast cancer and its activation is associated with cancer progression [39, 40], targeting cytosolic PINK1 might serve as a promising therapeutic approach against breast cancer in the future. Overall, the above studies describe the multifaceted nonmitochondrial roles of PINK1 in breast cancer, and the relationship between cytosolic PINK1 and breast cancer biology is still unclear and requires further study.

### Parkin in breast cancer

Parkin, an E3 ubiquitin ligase, is crucial for diverse cellular processes and is implicated in a variety of cancers. In breast cancer, the mRNA and protein levels of Parkin are frequently downregulated [35, 41–43]. Moreover, breast cancer patients with high Parkin expression tend to exhibit a lower histological grade, a lower proportion of triple-negative subtypes, decreased lymph node metastasis, and improved patient prognosis [42]. These results imply that Parkin acts as a tumor suppressor in breast cancer progression.

As reported, accumulating evidence indicates that Parkin exerts antitumor effects through various mechanisms in breast cancer. Early research revealed that Parkin has the potential to increase paclitaxel-microtubule interactions, thus enhancing the sensitivity of breast cancer cells to paclitaxel treatment [44]. Interestingly, Tay et al. revealed that Parkin enhances the mRNA and protein expression levels of cyclin-dependent kinase 6 (CDK6) and inhibits the proliferation and metastasis of MCF-7 cells both in vitro and in vivo [41]. However, the underlying mechanism by which Parkin regulates the transcript level of CDK6 remains obscure. Moreover, Liu et al. identified the classical role of Parkin as an E3 ubiquitin ligase and demonstrated its interaction with HIF-1α, facilitating HIF-1α degradation through ubiquitination [45]. This process effectively inhibits breast cancer metastasis [45]. Moreover, a similar relationship has been observed between Parkin and other proteins [35, 43, 46]. Miyahara et al. reported that Parkin directly interacts with BRCA1, leading to the ubiquitination of BRCA1 [35]. Given the significance of BRCA1 mutations in the development and progression of breast cancer [47, 48], these findings have potential prognostic and therapeutic implications for clinical breast cancer management. Phosphoglycerate dehydrogenase (PHGDH), the first rate-limiting enzyme of serine synthesis, is frequently upregulated in breast cancer and promotes tumor progression [49, 50]. However, Parkin was identified as a negative regulator of PHGDH by interacting with it and promoting its degradation through ubiquitination in breast cancer cells [43]. This finding emphasizes the role of Parkin in regulating cellular metabolism and underscores its potential significance in breast cancer pathogenesis.

### BNIP3, BNIP3L, and FUNDC1 in breast cancer

#### BNIP3

BNIP3, also known as BCL2/Adenovirus E1B 19 KDa Protein-Interacting Protein 3, is a member of the Bcl-2 proapoptotic family and is upregulated under hypoxic conditions [51]. An early study conducted IHC analysis of BNIP3 in 81 cases of breast ductal carcinoma in situ (DCIS) and 251 cases of invasive cancer [52]. The results had shown that the localization of BNIP3 was primarily detected in the cytoplasm rather than in the nucleus, and the expression level of cytoplasmic BNIP3 in DCIS and invasive cancer tissues was significantly greater than that in normal breast tissues [52]. Consistent with these findings, recent studies have reported that BNIP3 is upregulated in breast cancer specimens compared with normal specimens [53–55]. However, controversial results have been reported regarding the relationship between BNIP3 expression and its potential clinical significance in breast cancer patients. The nuclear expression of BNIP3 in invasive carcinomas was significantly correlated with decreased tumor size, low tumor grade, and estrogen receptor positivity [52]. However, no correlation was found between clinicopathologic variables and cytoplasmic BNIP3 expression in invasive carcinoma [52]. Moreover, breast cancer patients who are positive for BNIP3 expression exhibit lower rates of axillary lymph node metastasis [56], while high expression of BNIP3 in node-negative breast cancer patients is associated with a worse prognosis [57].

Accordingly, the biological function of BNIP3 in breast cancer is controversial. In the absence of external estrogen administration, overexpression of BNIP3 in ER-positive cells (MCF-7 cells) enhanced their tumorigenic potential in a mouse xenograft model, and there was a positive correlation between tumor size and BNIP3 expression intensity [58]. Consistent with these findings, in vitro experiments revealed that knockdown of BNIP3 expression in MCF-7 cells inhibited the malignant phenotypes of breast cancer cells under hypoxic conditions [55]. These results suggest that BNIP3 acts as an oncogene in MCF-7 cells, but the potential mechanism has not been fully identified. However, there is controversy concerning the role of BNIP3 in breast cancer progression. In an MMTV‐PyMT mouse mammary tumor model, loss of BNIP3 promoted tumor growth and accelerated lung metastasis in vivo [59]. Interestingly, compared with wild-type tumors, *BNIP3*-null tumors in this mouse model exhibited a loss of ER-α expression, and the loss of BNIP3 combined with high HIF‐1α levels was associated with a poor prognosis in TNBC patients [59]. Moreover, another study revealed that BNIP3 inhibits breast tumor growth and progression by activating apoptotic pathways [60]. Overall, BNIP3 exhibits complex and context-dependent functions in breast cancer biology, and the specific functions of BNIP3 in breast cancer progression need further investigation.

#### BNIP3L

Like BNIP3, BNIP3L (also called NIX) is a member of the Bcl-2 family of proteins and contains a BH3 domain [51, 61]. A previous study assessed the RNA level of BNIP3L and revealed that the expression of BNIP3L in breast cancer cells was similar to that in normal breast epithelial cells [62]. However, in breast tissue samples, researchers have shown that the level of BNIP3L is greater in primary breast cancers than in normal tissues [63]. Interestingly, another study involving IHC analysis of human breast tissues revealed that BNIP3L is abundantly expressed in the stroma of breast cancers but is largely absent from tumor cells [64]. Moreover, the ITGB4 protein derived from breast cancer exosomes has been shown to upregulate BNIP3L expression in cancer-associated fibroblasts (CAFs), thereby inducing BNIP3L-dependent mitophagy in CAFs and providing energy metabolites for breast cancer cells [65]. These findings indicate that BNIP3L may play a significant role in modulating tumor-stromal interactions.

In terms of the biological function of BNIP3L, Pedanou et al. reported that BNIP3 and BNIP3L, which are transcriptionally regulated by KDM3A, can promote anoikis in breast cancer cells [66]. In addition, another study revealed that BNIP3L is also transcriptionally activated by FOXO3A and sensitizes breast cancer cells to chemotherapy-induced apoptosis [67]. These results suggest the potential significance of BNIP3L in preventing cancer metastasis and enhancing chemotherapy efficacy in breast cancer patients.

#### FUNDC1

FUN14 domain-containing 1 (FUNDC1) is a newly identified mitochondrial outer membrane protein and its dysregulation has been implicated in various human diseases, including cancers [68]. Yang et al. reported that inhibition of FUNDC1 by miR-101 suppressed the proliferation and metastasis of TNBC in vitro and in vivo [69], suggesting that FUNDC1 acts as a tumor-promoting factor in TNBC. Similarly, another study revealed that the expression level of FUNDC1 in breast cancer tissues was significantly greater than that in normal breast tissues, and higher expression of FUNDC1 was related to worse disease progression in breast cancer patients [70]. Furthermore, they also reported that elevated FUNDC1 promotes Ca^2+^ flux into the cytoplasm, leading to the dephosphorylation of NFATC1. Consequently, this activation of NFATC1 stimulates BMI1 expression at the transcriptional level, thereby promoting the progression of breast cancer [70]. This result highlights the importance of FUNDC1-mediated signaling pathways in breast cancer progression beyond its role in mitophagy.

## Mitophagy in breast cancer progression

### Mitophagy in tumor growth and metastasis

Accumulating evidence suggests that the accumulation of intracellular reactive oxygen species (ROS) is associated with tumor promotion and progression [71]. However, mitophagy can remove dysfunctional mitochondria and reduce the production of ROS in tumor cells, thus exerting a tumor suppressor effect. For example, Deng et al. proposed that ULK1 depletion in breast cancer cells results in mitophagy deficiency, leading to excessive ROS production. This aberrant ROS accumulation subsequently activates the NLRP3 inflammasome and promotes breast cancer bone metastasis [72]. Consistently, another study provided further evidence supporting the role of BRCA1 as a tumor suppressor by regulating mitophagy [73]. Mechanistically, mitophagy is impaired in BRCA1-deficient cells through the blockade of mitochondrial fission, resulting in the accumulation of damaged mitochondria and increased levels of ROS [73]. This accumulation further triggers NLPR3 inflammasome activation and promotes breast cancer metastasis [73]. Consistent with these findings, Chourasia et al. demonstrated that mitophagy defects resulting from BNIP3 loss can also lead to the accumulation of dysfunctional mitochondria and increased levels of ROS, which in turn enhances the growth and metastasis of TNBC cells [59]. Therefore, reducing ROS production by restoring proper mitophagy may become a therapeutic strategy for treating breast cancer. Interestingly, growing evidence indicates that certain compounds and drugs exert antitumor effects on breast cancer through the activation of mitophagy [74–77]. These findings further highlight the significance of mitophagy modulation in breast cancer suppression.

However, mitophagy plays dual roles in cancer development in response to various stress conditions [15, 78]. In certain circumstances such as metabolic stress or nutrient deprivation, mitophagy can also promote tumor survival to adapt to unfavorable conditions [79, 80], thereby acting as a tumor-promoting mechanism in cancer development. There is evidence that the upregulation of MRPL52 promotes protective mitophagy through the PINK1/Parkin pathway, which helps breast cancer cells survive under hypoxic conditions [81]. Indeed, cancer cells may enter a state of dormancy to protect themselves against inhospitable microenvironments [82]. Interestingly, Vera-Ramirez et al. revealed the activation of mitophagy in dormant BC cells [83]. These studies suggest that mitophagy can serve as a protective mechanism for breast cancer cells against adverse microenvironmental conditions. Moreover, another study discovered that Mucin1 (MUC1), localized to the mitochondrial outer membrane, can promote PINK1-dependent mitophagy by interacting with the mitochondrial protein ATAD3A [84]. Moreover, the authors showed that inhibition of either MUC1 or mitophagy alone impedes tumor cell proliferation [84]. Remarkably, when these two approaches are combined, they exhibit even more effective inhibitory activity against breast tumor growth both in vivo and in vitro [84]. Therefore, targeting mitophagy has therapeutic potential in breast cancer for certain conditions.

### Mitophagy in metabolic reprogramming

In metabolically hostile environments, tumor cells can undergo metabolic reprogramming to ensure their survival despite limited oxygen and nutrient availability [85]. Given the central roles of mitochondria in cellular energy and redox homeostasis, a number of studies have investigated how mitophagy modulates metabolic reprogramming during breast cancer progression [86, 87].

Indeed, cancer cells exhibit a unique metabolic phenomenon known as the Warburg effect, where they preferentially utilize glycolysis for energy production despite the availability of oxygen [88]. Dysfunctional mitochondria and impaired mitophagy may compromise oxidative phosphorylation (OXPHOS) activity, leading to an increased reliance on glycolysis for energy production during cancer development. An associated study revealed that the activation of insulin-like growth factor 1 (IGF-1)/phosphoinositide 3-kinase (PI3K) signaling promotes BNIP3-mediated mitophagy in breast cancer [89]. This mechanism enhances the degradation of dysfunctional mitochondria by upregulating mitophagy, consequently preserving mitochondrial homeostasis to support OXPHOS activity [89]. Moreover, it has been shown that breast cancer cells with acquired resistance to an IGF-1 receptor tyrosine kinase inhibitor exhibit decreased mitophagy and increased dependence on glycolysis rather than OXPHOS for ATP production [89]. In agreement with this, another study revealed that deficiency in BNIP3‐dependent mitophagy leads to the accumulation of dysfunctional mitochondria, which exhibit increased ROS production [59]. These elevated levels of ROS subsequently promote HIF-1α activity, which further induces aerobic glycolysis and reduces OXPHOS activity in breast cancer cells [59]. Interestingly, the loss of BNIP3 is associated with malignant progression in TNBC, and treating *BNIP3*-null cells with 2‐deoxyglucose (2DG) to inhibit glycolysis has been demonstrated to effectively suppress breast cancer growth in vitro [59]. These findings suggest that targeting glycolysis could represent a highly promising anticancer therapeutic approach in breast cancer, especially when the OXPHOS pathway is already impaired by dysfunctional mitochondria.

Notably, excessive mitophagy can also lead to detrimental effects. Compelling evidence indicates that HIF-1α can promote the transcriptional activity of EBF1 [90]. Conversely, EBF1 interacts with HIF-1α and attenuates its activity by interfering with the activity of p300, which forms a negative feedback loop and maintains mitochondrial homeostasis in TNBC [90]. Intriguingly, the absence of EBF1 causes persistent activation of HIF-1α, resulting in pronounced mitophagy and a metabolic shift from glucose metabolism toward glycolysis in TNBC [90]. Consistent with these findings, Zou et al. revealed that Mdivi-1 could suppress mitophagy in MDA-MB-231 cells [91]. This inhibition leads to an increase in cellular OXPHOS capacity and a reduction in glycolytic activity, ultimately resulting in the suppression of growth in TNBC cells [91]. Moreover, previous studies have shown that Warburg-type metabolism is the most common metabolic phenotype in TNBC [92], suggesting that targeting abnormal metabolic pathways via mitophagy may represent a novel approach for inhibiting TNBC cell progression.

### Mitophagy in the tumor microenvironment (TME)

The TME involves a complex network of components, including primary tumor cells, various stromal and immune cells, the extracellular matrix, and cytokines [93]. Considerable progress has already been made in understanding the role of mitophagy in dynamically regulating the TME and its impact on tumor progression and treatment strategies.

Recent investigations have revealed a novel metabolic phenomenon called the reverse Warburg effect, in which CAFs undergo aerobic glycolysis to provide metabolic support for cancer cells [94, 95]. In the breast cancer stromal microenvironment, evidence suggests that cancer cells induce oxidative stress in adjacent CAFs [96, 97]. This oxidative stress then initiates mitophagy in CAFs, leading to the loss of functional mitochondria and a shift toward aerobic glycolysis [96, 97]. Consistent with these findings, a study revealed that ethanol exposure significantly downregulated Cav-1 expression while inducing mitophagy in CAFs [98]. This dysregulation in stromal fibroblasts further promotes the generation of ketone bodies, which serve as energy-rich substrates for breast cancer cells [98]. Notably, several additional studies reported that loss of Cav-1 in breast stromal fibroblasts is a functional biomarker of mitophagy in stromal cells under conditions of oxidative stress [97] and is associated with tumor-stromal metabolic coupling in the TME [99, 100]. Importantly, the absence of Cav-1 in stromal fibroblasts could be used to predict poor clinical outcomes in breast cancer patients [100–102]. In addition, Sung et al. elucidated an alternative mechanism by which breast cancer cells transport the ITGB4 protein via the exosome pathway. This process further induces BNIP3L-dependent mitophagy and glycolysis in CAFs [65]. Taken together, these studies highlighted the role of mitophagy in CAFs, which provides high-energy metabolites to tumors through aerobic glycolysis and promotes breast cancer progression [65, 96, 97], and that targeting mitophagy in CAFs could represent a promising therapeutic strategy for inhibiting breast cancer progression by disrupting metabolic interactions between CAFs and cancer cells.

Moreover, mitophagy in immune cells such as macrophages may affect their functions. For example, Zheng et al. demonstrated that urolithin A (UA) treatment activates PINK1/Parkin-driven mitophagy in tumor-associated macrophages, resulting in a decrease in stimulator of interferon genes (STING)-mediated inflammation and subsequently a reduction in the release of inflammatory factors such as IL-6 in the TME [103]. Mechanistically, UA inhibits TFEB ubiquitination-mediated degradation and enhances TFEB nuclear translocation in tumor-associated macrophages, thereby promoting the transcriptional activation of mitophagy-related genes regulated by TFEB [103]. This finding highlights the potential therapeutic significance of targeting mitophagy in immune cells as a strategy to modulate inflammation and reshape the TME, thus exerting antitumor effects on breast cancer.

Specifically, defective mitophagy within cells leads to the accumulation of ROS, which then activates inflammasome and contributes to TME remodeling [104]. Notably, several publications have described the significant roles of ROS in the breast tumor microenvironment [105, 106]; however, whether these ROS are directly derived from dysregulated mitophagy remains an open question. In the context of mitophagy, the evidence indicates that the absence of BRCA1 is associated with impaired mitophagy, resulting in the accumulation of damaged mitochondria and increased ROS levels [73]. Subsequently, this excessive ROS accumulation triggers the activation of the NLRP3 inflammasome and the secretion of mature cytokines such as interleukin-1β (IL-1β) within the TME, which facilitates breast cancer recurrence and metastasis [73]. In addition, Sun et al. applied a CCCP-loaded nanoplatform to induce excessive mitophagy in 4T1 cells [107]. Interestingly, during mitophagy, the authors observed the release of several endogenous DAMPs (such as ATP and HMGB1) and the expression of calreticulin (CRT) on the surface of 4T1 cells, which led to increased tumor-infiltrating lymphocytes (TILs) and elicits robust immune activation within the tumor microenvironment (TME) [107].

Overall, mitophagy plays a multifaceted role across different cellular components within the TME. This includes mediating metabolic reprogramming in CAFs, modulating immune cell function, regulating oxidative stress, and facilitating the release of DAMPs from tumor cells. Notably, exploring the therapeutic implications of targeting mitophagy within the context of TME reprogramming may open up innovative avenues for breast cancer treatment.

### Mitophagy in stem-like cells

Breast cancer stem cells (BCSCs) are a small subpopulation of cancer cells within breast tumors that exhibit stem cell-like properties, such as tumor onset, self-renewal, multidirectional differentiation, and treatment resistance [108]. Although previous research has explored the potential significance of mitophagy in several stem cell developmental processes [15, 109–111], the role and mechanism of mitophagy in maintaining stemness and regulating differentiation in BSCSs remain to be fully elucidated. Limited data indicate that miR-137 restored cell mitochondrial dysfunction by inhibiting FUNDC1-mediated mitophagy in BCSCs [112]. This intervention significantly attenuated the hypoxia-induced abnormal mitochondrial homeostasis, leading to inhibition of apoptosis and ultimately exerting a protective effect on the survival of BCSCs [112]. In accordance with this, another study demonstrated that overexpression of miR-1 increased the protein levels of LC3-II and PINK1 in BCSCs, thereby inducing mitophagy [113]. This mechanism is associated with the negative regulation of tumorigenesis in cancer stem cells [113]. However, since cancer stem cells are often characterized by their heightened reliance on mitochondrial function for energy production and self-renewal [114], disrupting the integrity of mitochondria through mitophagy induction may be a promising approach to impairing the survival and proliferation of BCSCs.

### Mitophagy in apoptosis, ferroptosis, and pyroptosis

**Fig. 2:**
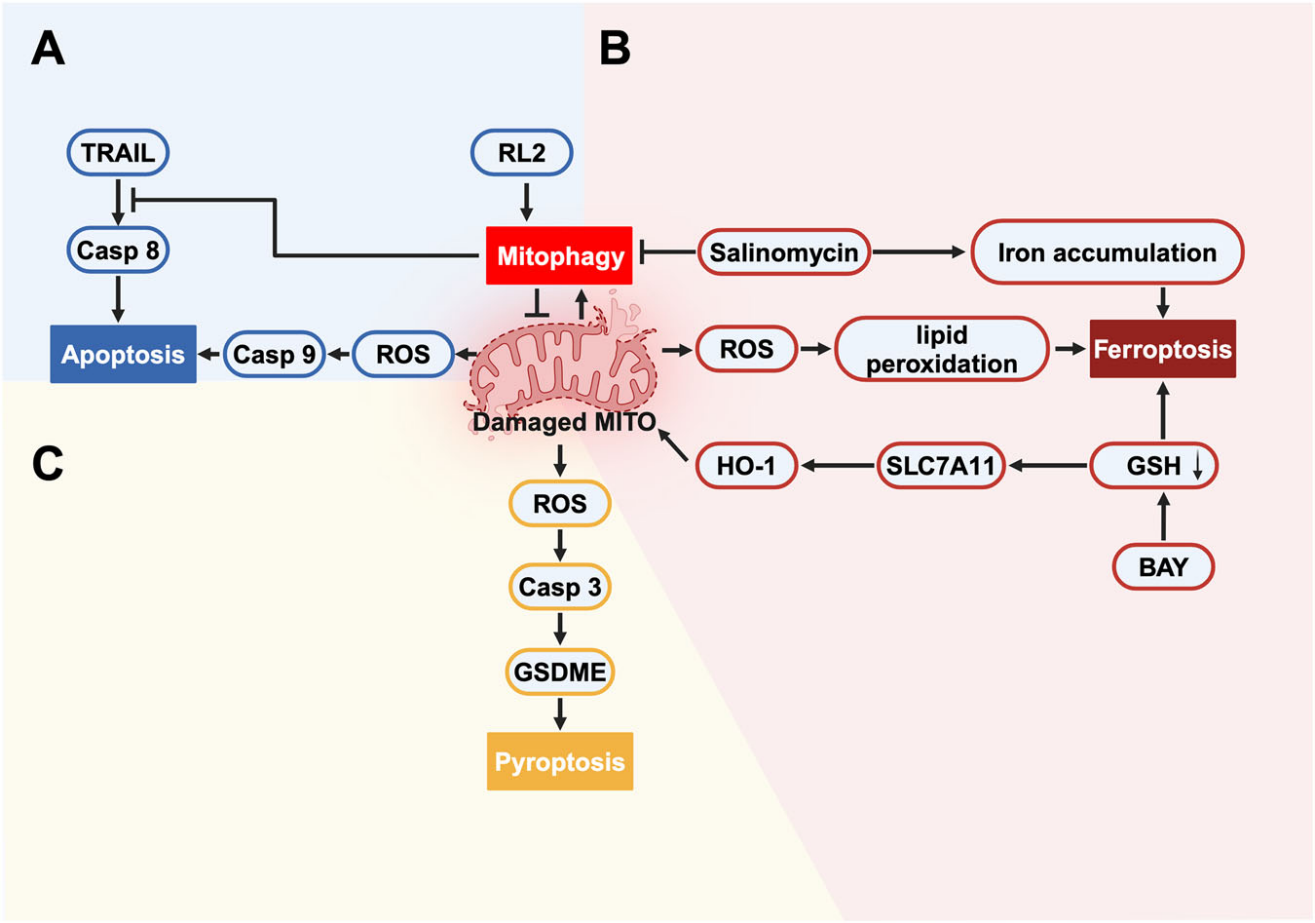
The interactions between three forms of RCD (apoptosis, ferroptosis, and pyroptosis) and mitophagy in breast cancer. Mitochondria are central regulators of both mitophagy and other forms of RCD. **A** By degrading damaged mitochondria, mitophagy prevents the release of ROS and keeps the cell from undergoing intrinsic apoptosis. Moreover, RL2-induced mitophagy blocks TRAIL-mediated extrinsic apoptosis by suppressing core proapoptotic regulators in breast cancer cells. **B** Treatment with salinomycin blocks the late stages of mitophagy, which leads to an accumulation of damaged mitochondria and makes breast cancer cells more prone to salinomycin-induced ferroptosis. In addition, certain ferroptosis-inducing agents, such as BAY, can induce ferroptosis through the Nrf2-SLC7A11-HO-1 pathway. Meanwhile, upregulation of HO-1 by BAY treatment can also lead to mitochondrial damage and activate mitophagy in breast cancer cells. **C** Accumulation of mitochondrial ROS (mtROS) can lead to oxidative stress, thereby activating the caspase 3-GSDME pyroptosis pathway. Therefore, mitophagy acts as a protective mechanism that prevents pyroptosis by clearing damaged mitochondria. Created with BioRender.com.

RCD, also known as programmed cell death, is the autonomous and orderly death of cells controlled by specific genes [4]. In the context of cancer, several forms of RCD, including apoptosis, ferroptosis, pyroptosis, and cuproptosis have been identified and shown to play crucial roles in cancer development [115]. Interestingly, recent studies indicate a potential interaction between mitophagy and other forms of RCD, which might contribute to stress responses and disease progression in breast cancer. Nonetheless, for simplicity, we will only focus on interactions between three forms of RCD (apoptosis, ferroptosis, and pyroptosis) and mitophagy (Fig. 2).

A specific link between mitophagy activity and apoptosis has been suggested by several studies [116–119]. As mentioned above, suppressing mitophagy increases the level of ROS within the cell, and increased ROS can serve as an indicator of intrinsic apoptotic processes [120, 121]. For example, Liu et al. revealed that FBP1 restrained mitophagy by suppressing the HIF-1α/BNIP3 pathway in breast cancer cells [122]. Accordingly, this inhibition of mitophagy results in cytotoxic ROS accumulation and eventually causes cancer cell apoptosis [122]. Consistently, another study on hypoxic breast cancer cells showed that Sirt3 can activate mitophagy by increasing the interaction of VDAC1 with Parkin. However, Sirt3 inhibition promotes the ROS-dependent proteasomal degradation of the Mcl-1 and survivin proteins, thereby triggering apoptosis in breast cancer cells [123]. Moreover, certain drugs (e.g., silibinin, polyphyllin I, and warangalone) have shown efficacy in inducing apoptosis in breast cancer cells to exert antitumor effects, but these effects are prevented by mitophagy [124–126]. Taken together, these data indicate that cancer cells might exploit mitophagy as a mechanism to evade apoptosis [119], and targeting mitophagy therefore represents a promising anticancer strategy against apoptosis-resistant breast cancer cells.

In addition to being related to the intrinsic (mitochondrial) apoptosis pathway, mitophagy also interacts with the extrinsic (cell death receptor) apoptosis pathway. The extrinsic pathway provides a rapid response to external signals, specifically tumor necrosis factor-alpha (TNF-α), Fas ligand (FasL), and TNF-related apoptosis-inducing ligand (TRAIL) [127]. Accordingly, RL2 (recombinant lactaptin 2) serves as a pro-mitophagy factor in breast cancer cells by interacting with TOM70 in mitochondria [128, 129]. However, the effects of RL2/TRAIL cotreatment exhibit opposite outcomes: under short-term stimulation, RL2-induced mitophagy can alleviate TRAIL-mediated extrinsic apoptosis, whereas under long-term costimulation, RL2 enhances TRAIL-induced cell death [128]. In addition, in combination with doxorubicin, RL2 can also promote doxorubicin-induced caspase-3/7 activity in breast cancer cells, thereby increasing the level of apoptosis and amplifying the antitumor effects of doxorubicin [129]. Therefore, through a well-considered combination of drugs affecting mitophagy and apoptosis, these two different types of programmed cell death can cooperate with each other and lead to a stronger antitumor response.

Ferroptosis, which is characterized by intracellular iron accumulation and lipid peroxidation, has been extensively investigated in cancer research in recent years [130]. Morphologically, cells undergoing ferroptosis display distinctive mitochondrial characteristics, including mitochondrial shrinkage, increased membrane density, and decreased or absent mitochondrial cristae [131]. Interestingly, several lines of evidence have shown crosstalk between these two forms of RCD [132–134]. For example, Yu et al. reported that the inhibition of O-GlcNAcylation results in extensive mitophagy, which helps release stored iron and renders cell lines more sensitive to ferroptosis [133]. In contrast, Lin et al. revealed that mitophagy could also alleviate cisplatin-induced ferroptosis through the ROS/HO-1/GPX4 axis [135]. Therefore, depending on the specific context, mitophagy can act as both an enhancer and a suppressor of ferroptosis. In breast cancer, researchers revealed that BAY 11–7085 (BAY) induces ferroptosis and suppresses cell viability through the Nrf2-SLC7A11-HO-1 pathway [136]. Additionally, HO-1 also mediates cell mitochondrial damage, leading to subsequent mitophagy [136]. This finding suggested that certain ferroptosis-inducing agents can impair mitochondrial function and activate mitophagy in breast cancer cells. In addition, Cosialls et al. demonstrated that mTOR inhibition could prevent salinomycin-induced ferroptosis in breast cancer stem cells [137]. Mechanistically, they observed that under salinomycin treatment, the late stages of mitophagy were inhibited, resulting in the accumulation of damaged mitochondria [137]. However, treatment with Torin, an mTOR inhibitor, was able to reactivate the late stages of mitophagy [137]. This reactivation of mitophagy helped overcome the accumulation of damaged mitochondria and protected breast cancer stem cells from salinomycin-induced ferroptosis [137]. In this context, mitophagy acts as a protective mechanism to remove damaged mitochondria, thus suppressing ferroptosis in breast cancer cells.

Pyroptosis, an inflammatory form of RCD mediated by GSDMs, is associated with tumor-associated inflammation [138]. Recently, several publications have documented the interplay between mitophagy and pyroptosis [139, 140]. In breast cancer, research has demonstrated that the mitochondrial protein UCP1 inhibits the metastasis and proliferation of TNBC cells in vitro and in vivo [141], and this effect has been attributed to the involvement of GSDME-mediated pyroptosis and PINK1/parkin-induced mitophagy [141]. This finding indicates a synergistic relationship between these two types of RCD in suppressing breast cancer. However, controversy exists regarding the protective cell survival pathways induced by mitophagy, which could hamper pyroptosis. To address this challenge, Deng et al. constructed a biomimetic nanoparticle loaded with pyroptosis-inducing nanoparticles (CG/RH-NPs) and mitophagy-inhibiting nanoparticles (CQ-NPs) [142]. In this system, CG/RH-NPs cause mitochondrial damage in 4T1 breast cancer cells, leading to the generation of substantial mitoROS, thereby activating the caspase 3-GSDME pyroptosis pathway [142]. However, the above process in turn activates compensatory mitophagy and attenuates the antitumor efficacy of CG/RH-NPs by eliminating damaged mitochondria and mitoROS [142]. Notably, the authors also highlighted the contribution of CQ-NPs with biomimetic nanoparticles, as they effectively block compensatory mitophagy, thereby amplifying the antitumor effect of pyroptosis [142]. Therefore, the above data indicate that addressing the complex interaction between mitophagy and pyroptosis pathways produced a novel combination therapy for breast cancer treatment.

Overall, understanding the intricate crosstalk between mitophagy and other forms of RCD provides valuable insights into how breast cancer cells maintain homeostasis, progress, and acquire drug resistance. In breast cancer treatment, promising results may be obtained when combining different types of RCD with mitophagy modulation.

## Mitophagy in breast cancer therapy

Chemotherapy offers significant benefits in reducing the risk of breast cancer recurrence and mortality. Notably, doxorubicin is a cornerstone of the standard chemotherapy regimen for the clinical management of breast cancer [143]. Recently, several clinical trials have investigated the role of chloroquine (CQ) and its derivative hydroxychloroquine (HCQ) in enhancing the efficacy of chemotherapy in various cancer types [144], which implies that disrupting the protective autophagic process has the potential to overcome chemoresistance. In terms of mitophagy, emerging evidence also shows that targeting mitophagy during chemotherapy represents a novel anticancer strategy for treating breast cancer. For example, Chang et al. reported that mitochondrial division inhibitor-1 blocks mitophagy initiation and prevents TNBC cells from clearing dysfunctional mitochondria, which enhances breast cancer cell sensitivity to doxorubicin [145]. Similarly, an additional study revealed that liensinine could inhibit mitophagy and lead to excessive accumulation of mitophagosomes, which sensitizes doxorubicin-induced cell death in breast cancer [146]. Consistently, Naso et al. demonstrated that PINK1/Parkin-dependent mitophagy could be activated in response to doxorubicin treatment in breast cancer and that mitophagy inhibition through miR-218-5p expression enhances the sensitivity of breast cancer cells to doxorubicin [147]. Although studies have shown that inhibiting mitophagy can enhance the sensitivity of breast cancer cells to doxorubicin, further investigation is needed to determine whether similar effects can be observed with other chemotherapeutic agents, such as paclitaxel.

Notably, immune checkpoint inhibitors (ICIs) are promising immunotherapies for some cancer types (such as non-small cell lung cancer or melanoma) and brings hope to breast cancer treatment, especially for TNBC. Previous studies have shown that programmed death-ligand 1 (PD-L1) is often upregulated in breast cancer cells as a mechanism of immune evasion, and PD-L1 blockers have emerged as promising therapeutic agents for the treatment of TNBC [148, 149]. Interestingly, Xie et al. revealed that mitophagy-mediated PD-L1 distribution in TNBC samples is correlated with the therapeutic response to ICIs combined with paclitaxel [150]. Specifically, their study revealed that paclitaxel treatment upregulates the level of ATAD3A, which suppresses PINK1 function in TNBC cells [150]. This suppression of PINK1 prevents the recruitment of PD-L1 to mitochondria by binding with PD-L1, thereby inhibiting PD-L1 degradation via mitophagy [150]. However, reduced PD-L1 mitochondrial localization leads to failure of mitophagy-dependent PD-L1 degradation, resulting in excessive accumulation of PD-L1 on the cell surface [150]. This increase in surface PD-L1 can reduce the efficacy of immunotherapy by promoting immune evasion in TNBC. Therefore, upregulating PD-L1 distribution in mitochondria through the PINK1-mediated mitophagy pathway might enhance the efficacy of anti-PD-L1 therapy for breast cancer.

In clinical practice, it has been shown that breast cancer patients with BRCA mutation could benefit from olaparib treatment. Accordingly, Arun et al. reported that mitophagy contributes to olaparib-induced cell death in BRCA1- and BRCA2-mutant breast cancer cell lines [151]. This finding suggests a potential additional mechanism by which olaparib exerts its anticancer effects beyond its role in inhibiting DNA repair. Moreover, the induction of mitophagy might elicit additive or synergistic effects of olaparib in BRCA-mutated breast cancer cells.

Radiotherapy is another important treatment for breast cancer. Zheng et al. constructed a novel fusion protein called TAT-ODD-p53, which consists of wild-type p53 combined with the TAT domain and the minimum oxygen-dependent degradation domain (ODD) of HIF-1α [152]. This innovative fusion protein was further confirmed to sensitize hypoxic breast cells to irradiation by inhibiting Parkin-mediated mitophagy [152]. Mechanistically, p53 can physically bind to Parkin and interfere with its translocation to mitochondria, thereby blocking mitophagy [152]. This finding suggested that mitophagy plays a protective role in cellular homeostasis under radiation. Nevertheless, another study proposed that PINK1-induced mitophagy, triggered by mitochondrial ROS (mitoROS), can initiate the sensitization of breast cancer cells to radiation [153]. Therefore, mitophagy also plays a dual role in radiation therapy for breast cancer treatment, acting as both a protective mechanism for cancer cell survival and a pro-death pathway that increases cancer cell sensitivity to radiation. However, the balance between the protective and damaging effects of mitophagy during the response to radiation presents a dilemma for further therapeutic intervention.

Meanwhile, recent studies have shown that several small-molecule chemicals and medications can regulate mitophagy in breast cancer cells, and provides a promising avenue for cancer therapy. Here, we summarize these agents in Table 1. However, when considering mitophagy as a new therapeutic avenue against breast cancer, it is crucial to fully understand its dual effects on tumors. Generally, basic or moderate mitophagy degrades the damaged mitochondria, which supports cellular energy production and promotes mitochondrial homeostasis. This protective mechanism provides a survival advantage to tumor cells under various stress conditions. In contrast, excessive or persistent mitophagy causes mitochondrial depletion and impairs energy metabolism, which leads to the death of tumor cells [154]. For example, Qiu et al. demonstrated that the absence of EBF1 triggers pronounced mitophagy in TNBC cells, which results in robust metabolic reprogramming, mitochondrial dysfunction, and eventual cell death [90]. Besides, several studies have employed excessive mitophagy as a therapeutic strategy against breast cancer. It has been shown that flubendazole upregulates DRP1 expression and results in excessive mitophagy through PINK1/parkin signaling in breast cancer cells [74]. This excessive mitophagy leads to mitochondrial damage and dysfunction, subsequently inhibiting the proliferation and migration of breast cancer cells [74]. Consistently, acid ground nano-realgar processed product could also suppress breast cancer cell proliferation by activating the p53/BNIP3/NIX mitophagy pathway [76]. Moreover, Shen et al. reported that Guangsangon E inhibited TNBC cell growth and induced non-apoptotic cell death in vitro and in vivo, with its effects strongly associated with the activation of mitophagy [155]. Therefore, excessive mitophagy disrupts mitochondrial homeostasis and impairs energy production, ultimately contributing to the death of tumor cells.

**Table 1:** Modulation of mitophagy in breast cancer-currently available pharmaceutical agents.

Agents	Cell lines	Pathways	Effects	Years
Liensinine [146]	MDA-MB-231	Inhibit autophagosomal-lysosomal fusion	Inhibit mitophagy	2015
Olaparib [151]	BRCA mutant cells	Unknown	Induce mitophagy	2015
Polyphyllin I [125]	MDA-MB-231, MCF-7	PINK1-mediated	Induce mitophagy	2017
Flavonoid TL-2-8 [156]	MDA-MB-231, MDA-MB-468	PINK1-mediated	Induce mitophagy	2017
Chalcomoracin [157]	MDA-MB-231	PINK1-mediated	Induce mitophagy	2018
Vincristine [77]	MDA-MB-231	Hsp70 acetylation	Induce mitophagy	2019
B5G1 [158]	MCF-7/ADR	PINK1-mediated	Induce mitophagy	2019
Silibinin [124]	MDA-MB-231, MCF-7	PINK1-mediated	Induce mitophagy	2020
Ginsenoside Rh2 [159]	Senescent MCF-10A	PINK1-mediated	Induce mitophagy	2020
RL2 [128, 129]	MDA-MB-231, BT-549, T-47D	Upregulate BNIP3, BNIP3L, and PINK1	Induce mitophagy	2020, 2023
Metformin Prodrugs [160]	MDA-MB-231	Unknown	Induce mitophagy	2021
Warangalone [126]	MDA-MB-231, MCF-7	PINK1-mediated	Induce mitophagy	2021
Flubendazole [74]	MDA-MB-231, MCF-7	PINK1-mediated	Induce mitophagy	2022
Taloxifene [161]	MCF-7	PINK1-mediated	Induce mitophagy	2022
Guangsangon E [155]	MDA-MB-231, MDA-MB-468, MDA-MB-453	PINK1-mediated	Induce mitophagy	2022
Gramicidin A [162]	MCF-7	Unknown	Induce mitophagy	2022
Cepharanthine [163]	MDA-MB-231	PINK1-mediated	Inhibit mitophagy	2022
Artesunate [164]	MDA-MB-231	PINK1-mediated	Induce mitophagy	2022
Acid ground nano-realgar processed product [76]	MDA-MB-435S	PINK1-mediated	Induce mitophagy	2023
Kaempferol [75]	MCF-10AT	PINK1-mediated	Induce mitophagy	2023
Costunolide [165]	MCF-7	PINK1-mediated	Induce mitophagy	2023
Copper complex CPT8 [166]	MDA-MB-231	PINK1 and BNIP3-mediated	Induce mitophagy	2023
Baicalein [167]	MDA-MB-231	PINK1-mediated	Induce mitophagy	2023
Ru-TPE-PPh complex [168]	MDA-MB-231	PINK1-mediated	Induce mitophagy	2024
Idebenone [169]	MDA-MB-231, MDA-MB-468	Unknown	Induce mitophagy	2024

## Conclusions and future perspectives

The physiological process and molecular mechanism of mitophagy have been extensively investigated in recent years, particularly in the context of cancer and neurodegenerative diseases [11]. In this review, we first summarize the expression patterns and functions of mitophagy-related molecules in breast cancer. Notably, a comprehensive understanding of these molecules will assist in identifying the occurrence of mitophagy in breast cancer cells and may facilitate the discovery of new therapeutic targets in breast cancer therapy. Besides, we present evidence of mitophagy on breast cancer progression from multiple perspectives, including tumor growth and metastasis, metabolic reprogramming, the tumor microenvironment, and stem-like cell characteristics. These findings will offer valuable insights into the multifaceted roles of mitophagy in breast cancer progression. Moreover, the interactions between mitophagy and other forms of RCD offer promising evidence for developing combined therapeutic strategies for breast cancer treatment.

However, compared with the issues that have been resolved, there is still a gap in the research on translating mitophagy-based mechanistic research into effective breast cancer therapy. Does mitophagy increase or decrease the risk of developing breast cancer? After tumor initiation, how does mitophagy govern the fate of breast cancer cells (promoting or inhibiting tumors) at different stages, different molecular types, and different tumor microenvironments of breast cancer? How can we modulate mitophagy to enhance the efficacy of chemotherapy, radiotherapy, or immunotherapy in breast cancer treatment? As alterations in mitophagy are also related to the development of other diseases, such as neurological disorders, how can we weigh and avoid adverse effects when modulating mitophagy as a therapeutic approach in breast cancer treatment? In clinical practice, some anticancer drugs exert antitumor effects by inducing apoptosis, ferroptosis or pyroptosis; however, breast cancer cells can exploit protective mitophagy mechanisms to survive and develop drug resistance. Therefore, the precise interaction between mitophagy and RCD requires further exploration.

**Fig. 3:**
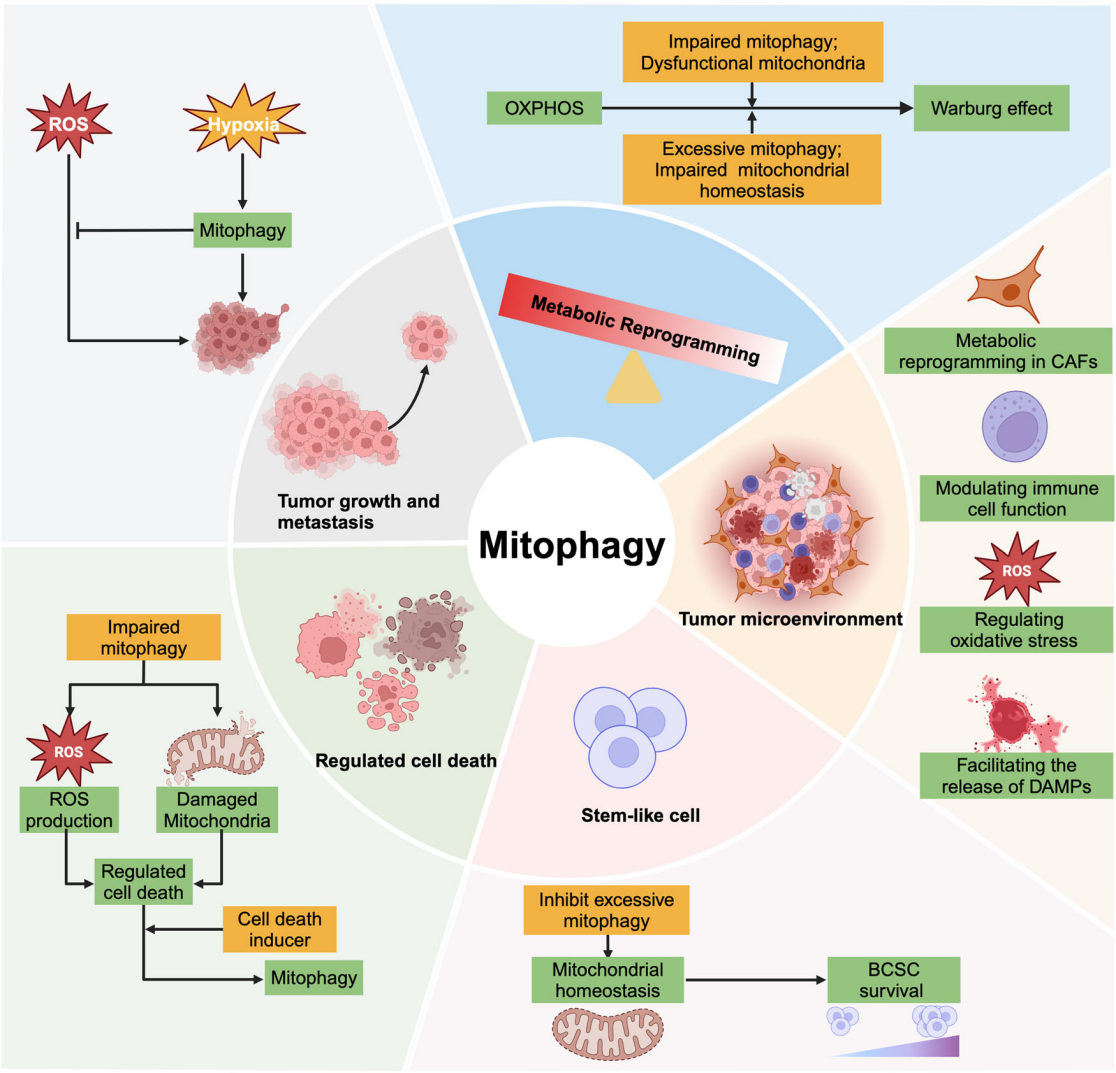
The implication of mitophagy in breast cancer progression. Modulating mitophagy can profoundly impact the progression of breast cancer through tumorigenesis, metastasis, metabolic reprogramming, the tumor microenvironment, stem-like cell characteristics, and regulated cell death. Created with BioRender.com.

In summary, modulating mitophagy can profoundly impact the progression of breast cancer through tumorigenesis, metastasis, metabolic reprogramming, the tumor microenvironment, stem-like cell characteristics, and RCD (Fig. 3). Moreover, modulating the process of mitophagy might offer novel therapeutic avenues for future breast cancer treatments. Notably, in the context of different stages, different molecular types, and different tumor microenvironments of breast cancer, the effects induced by mitophagy may be contradictory, and a comprehensive understanding of the full spectrum of mitophagy effects is required to develop efficient strategies for breast cancer treatment.
